# Estimating true hospital morbidity of complications associated with mumps outbreak, England, 2004/05 

**DOI:** 10.2807/1560-7917.ES.2016.21.33.30320

**Published:** 2016-08-18

**Authors:** CF Yung, M Ramsay

**Affiliations:** 1 Infectious Disease Service, Department of Paediatrics, KK Women’s and Children’s Hospital, Singapore; 2 Immunisation, Hepatitis and Blood Safety Department, Public Health England, London, United Kingdom

**Keywords:** England, mumps, measles-mumps-rubella (MMR) vaccine, outbreaks, public health policy, modelling

## Abstract

Mumps outbreaks in highly vaccinated populations continue to be reported globally. Therefore, quantifying the burden of mumps morbidity accurately will be necessary to better assess the impact of mumps vaccination programmes. We aim to estimate the true morbidity resulting from mumps complications in terms of hospitalised orchitis, meningitis, oophoritis and pancreatitis in England during the outbreak in 2004/05. This outbreak in England led to a clear increase in hospitalisations coded to mumps for complications of orchitis in those born in the 1970s and 1980s and possibly for meningitis in those born in the 1980s. A simple statistical model, based on analysing time trends for diagnosed complications in hospital databases with routine laboratory surveillance data, found that the actual morbidity was much higher. There were 2.5 times (166 cases) more mumps orchitis cases in the 1970s cohort and 2.0 times (708 cases) more mumps orchitis cases in the 1980s cohort than complications coded to mumps in hospital databases. Our study demonstrated that the mumps outbreak in England 2004/05 resulted in a substantial increase in hospitalised mumps complications, and the model we used can improve the ascertainment of morbidity from a mumps outbreak.

## Introduction

Mumps is an acute viral infection which can present with fever, headache and swelling of the parotid glands (unilateral or bilateral). It can be asymptomatic in around 30% of children [1]. Reported complications of mumps infection include orchitis, aseptic meningitis, oophoritis, pancreatitis, encephalitis and permanent unilateral deafness [1-3].

The measles, mumps, rubella (MMR) vaccine was introduced into the immunisation programme in the United Kingdom (UK) in October 1988 as a single dose for children aged 12 to 15 months. In 1996, to provide additional protection against all three infections, a second dose was added to the schedule. In the first decade after MMR vaccine was introduced, rates of reported and confirmed mumps infection fell to extremely low levels in the UK (42 confirmed mumps cases in 1998). Since 1998, however, there have been a number of mumps outbreaks in adolescents culminating in a national epidemic in 2004 and 2005, affecting many universities and colleges across England and Wales. The age group mainly affected were those born before 1988, who had not been offered routine childhood MMR vaccination and who had avoided mumps exposure because of high coverage in younger children [4]. Although MMR coverage in two-year-old children was only ca 80%, the outbreak dynamics did not result in significant transmission in young children [5,6]. The introduction of a second MMR dose for pre-schoolers in 1996 could be a factor which may have contributed to this [4]. The number of laboratory-confirmed mumps cases rose from 500 in 2002 to 1,541 in 2003 to 8,129 in 2004 and 43,378 in 2005 [5].

Routine data on hospitalisations can provide information on complications of mumps if the diagnosis of mumps is obvious and coded correctly on discharge. Delayed or missed diagnoses have been shown to occur, particularly in the absence of a history of parotid swelling [7]. This may lead to an underestimation of complications attributable to mumps. As mumps outbreaks in highly vaccinated populations continue to be reported globally, quantifying the burden of mumps morbidity accurately will be necessary to better assess the impact of mumps vaccination programmes [7-16]. In this paper, we used regression analysis to assess the contribution of laboratory-confirmed mumps cases to the hospitalisations for orchitis, meningitis, oophoritis and pancreatitis in each birth cohort. Other common infections associated with meningitis were used as an additional parameter in the regression model for meningitis only. This enabled us to estimate the number of hospitalised orchitis, meningitis, oophoritis and pancreatitis attributable to the increase in mumps during the outbreak.

Therefore, the aim of the study was to estimate the true morbidity resulting from mumps complications in terms of hospitalised orchitis, meningitis, oophoritis and pancreatitis in England during the mumps outbreak in 2004/05.

## Methods

### Data source

#### Selection of birth cohort cases and study period

For the purpose of this study, data were retrieved from 1 April 2002 to 31 March 2006 from all data sources. As the mumps outbreak mainly involved young adults, those born between 1970 and 1999 were included and cases were divided into three birth cohort decades for analysis: 70s (1970–79), 80s (1980–89) and 90s (1990–99). These birth cohorts were affected largely because they were not eligible for vaccination and had avoided exposure during childhood. As the outbreak occurred over a short time frame, the cohorts provide a suitable proxy for age while at the same time keeping the modelling analysis less complex.

#### Laboratory-confirmed mumps cases

Confirmed mumps infections are reported by laboratories in England to Public Health England (PHE) (formerly the Health Protection Agency). Clinicians who diagnose mumps are also required by statute to notify the responsible public health officer for the local authority, usually a consultant in Communicable Disease Control. Since 1995, all notified cases of mumps have been followed by an offer of oral fluid testing for IgM at PHE. The oral fluid test had been shown to have a sensitivity of 90.3% and specificity of 97.6% [17]. A high proportion of notified cases provided samples and were tested using this method (50–80%) [18]. Some cases were confirmed on serum only using commercial mumps IgM assays. Cases confirmed by testing for IgM in oral fluid or in serum were used to provide data on trends in the 70s and 90s cohort. In 2005, during the peak of the outbreak, mumps oral fluid testing was temporarily suspended in those born between 1981 and 1986. Therefore, to avoid any bias resulting from change in testing, only mumps confirmed by serum IgM testing was used to derive trends for the 80s cohort in our model.

In summary, cases were defined as individuals born between 1970 and 1999 with a diagnosis of laboratory-confirmed mumps via either IgM in oral fluid or serum from 1 April 2002 to 31 March 2006.

#### Hospital admissions database

Hospital Episode Statistics (HES) capture all admissions (including day admissions) to National Health Service (NHS) hospitals in England. The diagnoses recorded at the time of discharge are coded using the International Classification of Diseases-10 (ICD-10). A minimum dataset for all admissions with any of the following codes was extracted: B26 (mumps), N45 (orchitis and epididymitis), A87 (viral meningitis), N70 (oophoritis) and K85 (acute pancreatitis) [19]. The anonymised HESID field, which is a unique ID generated from NHS Number, local patient identifier, postcode, sex and date of birth was used to link episodes from the same individual admitted over the period [20]. Length of hospital stay is calculated from days between the admission date and the discharge date. 

#### Non-mumps meningitis trend data

For the viral meningitis model only, to adjust for the possible effect from non-mumps-related causes of meningitis, we included as an additional independent variable trends of laboratory-confirmed infections with other viruses known to be commonly associated with meningitis. The trend information used for not mumps-related causes of meningitis was derived from temporal data on confirmed meningitis cases due to coxsackievirus A and B, echovirus or untyped enterovirus during the study period. Because of the small numbers involved, this was included in aggregate form and not broken down by birth cohort.

### Statistical analysis

We developed least square regression models which associated hospital records of orchitis, meningitis, oophoritis and pancreatitis with parameters of laboratory-confirmed mumps cases and time. The formula for hospital records of complication Y in a four-week period j was:

Y_j_ = C + α L_j_ + γ_j_

where Y is the total number of recorded complications in the HES database, _j_ is unit time in intervals of four weeks, C is a constant representing background cases of complication Y attributable to non-mumps causes, α is the coefficient for laboratory-confirmed mumps infection, L is the number of laboratory-confirmed mumps and γ is the coefficient for unit of time (four weeks). We included γ to factor in age trends of mumps morbidity. Specifically for meningitis, laboratory-confirmed meningitis cases not due to mumps were included as an extra parameter in the equation.

The values of α were estimated by least square regression in Microsoft Excel version 11. The final model only included statistically significant explanatory parameters while non-significant parameters were dropped. The least significant parameter was removed first and one at a time. A parameter was only retained in the model if α remained significantly (p < 0.05) associated with hospital record of complication. The model was also eyeballed to ensure that the fit to actual data was reasonable, taking into consideration the goodness-of-fit of the model as denoted by R^2^.

The statistical models for each birth cohort and complication generated a value for the number of hospital records of complication associated with a single laboratory-confirmed case of mumps. This was used to estimate the morbidity attributable to the mumps outbreak in each birth cohort and complication by the sum of α L_j_ during the study period from the equation above, which is denoted by:

∑ α L_j_

Complications and birth cohorts for which the association with the parameter laboratory-confirmed mumps was not statistically significant (not p < 0.05) were dropped from the final model. In such a scenario, the model estimated no complications attributable to the mumps outbreak.

The method has been used in a similar way to investigate hospital admissions due to rotavirus, the contribution of respiratory syncytial virus to bronchiolitis and pneumonia-associated hospitalisation and to evaluate the contribution of serogroup C meningococcal infections to clinically diagnosed cases of meningitis and septicaemia [21-23].

## Results

### Laboratory-confirmed infections

**Figure 1:**
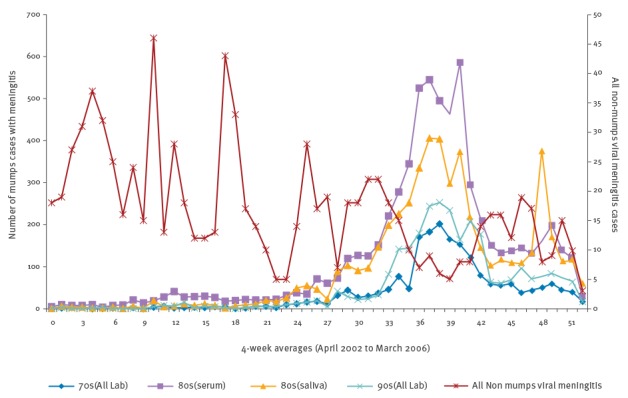
Four-weekly number of laboratory-confirmed mumps^a^ (n = 16,549) and non-mumps (n = 903) viral meningitis infections, England, April 2002–March 2006

^a^ Mumps infections stratified by birth cohort (and laboratory sample type for 80s cohort only).

The timing of the mumps outbreak in 2004/05 was apparent in laboratory-confirmed mumps cases in all three birth cohorts, particularly those born in the 80s cohort (Figure 1). Cases confirmed by oral fluid (saliva) in the latter cohort declined slightly earlier than in the other birth cohorts because of the restriction on oral fluid testing in this age group. Trends over time in the cases confirmed by serum testing in the 80s cohort, however, were similar to overall trends in the other cohorts. There were 900 laboratory-confirmed infections due to coxsackievirus A and B, echovirus and untyped enterovirus between April 2002 and March 2006. The trend in non-mumps causes of meningitis fluctuated over time, decreasing during the mumps outbreak period.

### Mumps-coded hospital admissions for orchitis, meningitis, oophoritis or pancreatitis (HES database)

Between April 2002 and March 2006, there were a total of 2,284 hospitalisations with a diagnostic code for mumps among those born between 1970 and 1999. Of these, 950 (42%), 143 (6%), 138 (6%) and 0 (0%) also had a second diagnosis code for orchitis, meningitis, pancreatitis and oophoritis, respectively. For hospitalised mumps orchitis, the 80s cohort contributed the largest numbers with 811 cases, while the 90s cohort was least represented with only 26 cases. The average length of stay for hospitalised mumps was shortest in the 90s cohort with 1.3 days, compared with 2.2 days and 1.9 days in the 70s and 80s cohorts, respectively. The pattern of highest burden in the 80s cohort and lowest burden in the 90s cohort was similar for mumps meningitis and pancreatitis but at a smaller scale due to the smaller number of such complications (Table 1).

**Table 1:** Hospital admissions of orchitis, meningitis, oophoritis and pancreatitis cases (n=27,133) and cases coded as mumps (n=1,231), by birth cohort, England, April 2002–March 2006

Mumps complication	Birth cohort			
	70s	80s	90s	Total
Orchitis				
Hospital cases	4,623	5,559	2,265	12,447
Hospital cases coded as mumps	113	811	26	950
Meningitis				
Hospital cases	1,978	1,787	294	4,059
Hospital cases coded as mumps	16	118	9	143
Oophoritis				
Hospital cases	924	709	40	1,673
Hospital cases coded as mumps	0	0	0	0
Pancreatitis				
Hospital cases	6,025	2,585	344	8,954
Hospital cases coded as mumps	13	114	11	138

### Non-mumps-coded hospital admissions for orchitis, meningitis, oophoritis and pancreatitis (HES database)

Between April 2002 and March 2006, there were a total of 12,447 hospital admissions for orchitis, 4,059 hospital admissions for meningitis, 1,673 hospital admissions for oophoritis and 8,954 hospital admissions for pancreatitis in patients born between 1970 and 1999 (Table 1).

**Figure 2:**
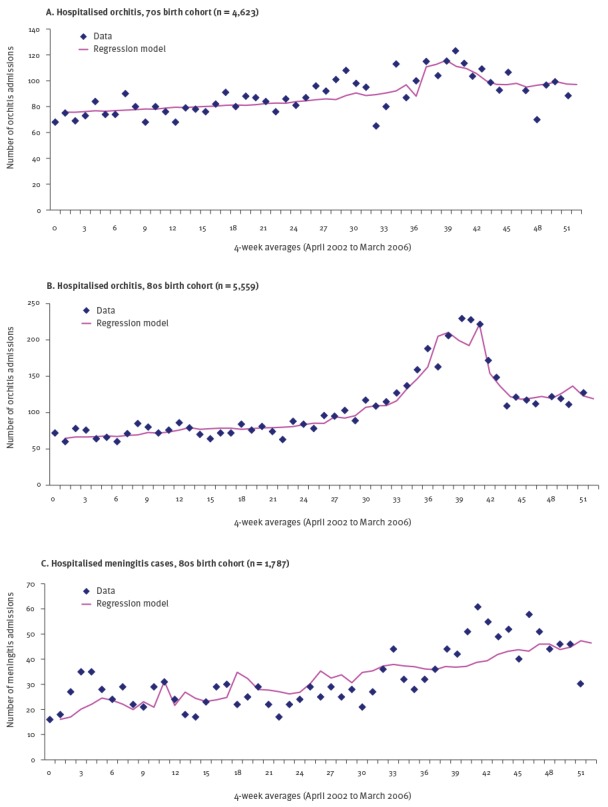
Regression model of hospitalised orchitis (A and B) and hospitalised meningitis cases (C), England, April 2002–March 2006

The number of hospital admissions for orchitis in the 70s and 80s birth cohorts peaked in 2004/05 coinciding with the peak of the mumps outbreak (Figure 2). Looking at the raw data, there also appeared to be a slight increase in hospitalisations for meningitis cases at this time in people born in the 80s (Figure 2C). However, the model did not demonstrate any statistically significant increase over the period. None of the other hospital records of orchitis, meningitis, oophoritis and pancreatitis by birth cohort showed any obvious spikes coinciding with the increase in laboratory confirmed mumps cases.

### Modelling to estimate true morbidity associated with mumps outbreak

The regression model was produced for hospitalisation with orchitis, meningitis, oophoritis and pancreatitis for the three birth cohorts (70s, 80s and 90s). Only the models for orchitis in the 70s and 80s cohort were statistically significant for all parameters. The models found 2.5 times more mumps orchitis in the 70s cohort (166 cases) and 1.9 times more mumps orchitis in the 80s cohort (708 cases) when compared with the number of cases in each cohort that were coded as mumps in hospital databases (HES). Apart from the two outcomes above, all other outcomes had no excess morbidity attributable to the mumps outbreak according to the model generated (Table 2).

**Table 2:** Orchitis and meningitis morbidity in hospitalised mumps cases attributable to mumps outbreak, by birth cohort, England, 2004/05 (n =1,798)

Birth cohort	Orchitis			Meningitis		
	70s	80s	90s	70s	80s	90s
Intercept (C)	75.13	63.44	28.49	29.4	10.47	5.65
Coefficient for mumps case (α)	0.14 (0.08–0.21)	0.23 (0.20–0.27)	^a ^	^a ^	^a ^	^ a^
Coefficient for unit time (γ)	0.32 (0.10–0.54)	0.5(0.18–0.92)	0.58 (0.44–0.73)	^a ^	0.64 (0.47–0.81)	^a ^
Coefficient for non-mumps meningitis	NA	NA	NA	0.50 (0.25–0.75)	0.31 (0.04–0.59)	^a ^
R^2^	0.59	0.88	0.57	0.25	0.56	0
Estimated cases attributable to mumps (∑ α L_j_)	279 (193–511)	1,519 (1,366–1,870)	0	0	0	0

NA: not applicable.

^a^ Parameter dropped from model as not significant.

## Discussion

The 2004/05 mumps outbreak in England led to an increase in hospitalisations coded as mumps orchitis in those born in the 1970s and 1980s and possibly those coded as mumps meningitis in those born in the 1980s. Our regression models echo these findings but found that the true burden of hospitalised mumps orchitis was 2–2.5 times greater than the number of cases actually coded as mumps in hospital databases. There was no obvious increase in other complications or age cohorts coinciding with the outbreak but there was a suggestion of higher numbers of mumps-coded pancreatitis (114 cases) in hospital records in those born in the 1980s.

During the years of low mumps incidence following introduction of the MMR vaccine, mumps morbidity due to orchitis, meningitis, pancreatitis and oophoritis was rare. The resurgence of mumps despite high coverage with two-dose MMR in many countries may require improved monitoring of mumps complications as well as may require consideration of new strategies. Mumps orchitis was the most common reason for hospitalisation, accounting for 42% of all hospitalised mumps cases, similar to large outbreaks reported from the Netherlands and from Jewish communities in the United States and Israel [7,15,24]. The high numbers of mumps orchitis in current mumps outbreaks may be attributed to the high attack rates in adolescents and young adults. Although mumps orchitis has been shown to cause acute azoospermia and oligospermia, the potential of mumps orchitis to lead to infertility in post-pubertal males remains unclear [25-29].

Our model demonstrated the limitation of using hospital surveillance records alone to study admissions attributable to mumps. To improve the quality of the data, clinicians would need to actively seek a history of mumps in patients who present with orchitis, meningitis and pancreatitis, and to record the history of recent mumps in the discharge summary. Better recording, however, is unlikely to fully resolve the issue of underascertainment of mumps complications as many patients may not have been aware that they have had mumps. Based on our model, the true burden of mumps orchitis would be 2 or 2.5 fold higher compared with cases actually coded as mumps in hospital databases during a mumps epidemic. However, even this level is likely to be a minimum since we have only looked at hospitalised morbidity. Nevertheless, our findings should provide a quantification to better estimate the true burden of hospitalised mumps morbidity. This has important implications for improving vaccination uptake at the frontline as well as describing the overall impact of mumps vaccination as the programme evolves.

Analysis of the hospitalised mumps population showed that the number of mumps complications varied between birth cohorts. Overall, those born in the 1990s had fewer complications and a shorter mean hospital stay (by an average of 0.75 days) compared with the other birth cohorts. Regression modelling identified a similar pattern with higher morbidity from orchitis in those born in the 1970s and 1980s compared with the 1990s birth cohort. It is unlikely that this is simply due to a difference in the number of mumps cases during the outbreak. Laboratory surveillance data suggest that the number of confirmed cases was similar in the 1970s and 1990s cohorts. A possible explanation is the protective effect of MMR vaccination against complications. Previously, we found that MMR vaccination reduces the risk of hospitalisation, orchitis and meningitis despite vaccine failure [30]. A similar protective effect of MMR vaccine especially against mumps orchitis have also been found in United States, the Netherlands and Israel [15,24,31]. In England, the 90s cohort, unlike the other cohorts, were likely to have received at least one or more doses of MMR as MMR vaccine was only introduced into the national programme in October 1988.

The model was unable to detect an increase in hospital morbidity for meningitis, oophoritis or pancreatitis in the affected cohorts. This is consistent with the observed lack of oophoritis coded as mumps but at odds with the increase in pancreatitis with a mumps code (138 cases) in routine hospital databases. Moreover, the statistical model was not able to detect a spike in hospitalised meningitis in the 80s cohort coinciding with the mumps outbreak. This highlights the limitation of the model and suggests that the burden of morbidity from the mumps outbreak is likely to be a minimum estimate. Other limitations included the assumption that all the parameters accounting for the reported variation in four-weekly numbers of hospitalisations were included in the model. In addition, we could only investigate complications that resulted in acute hospital admissions and we could not investigate long-term sequelae such as deafness resulting from mumps.

## Conclusion

Our study showed that the mumps outbreak in England 2004/05 resulted in a substantial increase in mumps complications of orchitis, pancreatitis and possibly meningitis with subsequent hospitalisations. We have shown that analysing time trends for all diagnoses of complications in hospital databases with routine laboratory surveillance data in a simple statistical model can improve the ascertainment of morbidity from a mumps outbreak. This method increased the morbidity due to mumps-related orchitis hospitalisations by a factor of 2 or 2.5 when compared with those coded as mumps alone.
